# Long-term costs of post-restorations: 7-year practice-based results from Germany

**DOI:** 10.1007/s00784-020-03529-5

**Published:** 2020-08-28

**Authors:** Falk Schwendicke, Enno J. Kramer, Joachim Krois, Hendrik Meyer-Lueckel, Richard Johannes Wierichs

**Affiliations:** 1 Department of Oral Diagnostics, Digital Health and Health Services Research, Charité - Universitätsmedizin Berlin, Berlin, Germany; 2 Department for Operative and Preventive Dentistry, Charité – Universitätsmedizin Berlin, Aßmannshauser Str. 4-6, 14197 Berlin, Germany; 3 Private Practice, Norden, Germany; 4 Department of Restorative, Preventive and Pediatric Dentistry, zmk bern, University of Bern, Bern, Switzerland; 5 Department of Biohybrid & Medical Textiles, Institute of Applied Medical Engineering, RWTH Aachen University, Aachen, Germany

**Keywords:** Dental, Endodontics, Glass fiber, Health economics, Metal, Posts, Restorations

## Abstract

**Objectives:**

We evaluated the initial and follow-up treatment costs of different post-restorations in a practice-based German healthcare setting.

**Methods:**

A total of 139 incisors, canines, or premolars received post-restorations placed by eight general dental practitioners in Germany, and were followed over a mean ± SD 7.1 ± 4.5 years. Preformed metal (MP, *n* = 68), glass-fiber (GF, *n* = 28), or cast post-and-core buildups (MC, n = 23) had been used to retain crowns or bridge anchors. Preformed metal and glass-fiber had also been used to retain directly built up post-retained composite restorations (PC, *n* = 20). Material and treatment costs for the initial post-restorations as well as restorative, endodontic, or surgical re-treatments were estimated from a public-payer-perspective in Germany. Associations between total and annualized total costs and covariates were assessed using generalized linear modeling. The study was registered in the German Clinical Trials Register (DRKS-ID: DRKS00012938).

**Results:**

MC showed highest initial treatment costs (557.23 Euro), but the least re-treatments (6/23, 26%), while PC showed lowest initial costs (203.52 Euro) but the most re-treatments (11/20, 55%). Costs for MP/GF post-crowns were initially similarly costly (496.47/496.87 Euro), and both also showed similar re-treatments (35%/36%). The overall annual failure rate was 5.2% (MC: 3.5%, MP: 4.6%, GF: 5.3%, PC: 10.3%). Including costs for the resulting re-treatments, mean total costs were 591.66 Euro (MC), 548.31 Euro (MP), 526.37 Euro (GF), and 361.81 Euro (PC). Annualized total costs were 171.36 Euro (MC), 141.75 Euro (MP), 146.12 Euro (GF), and 135.65 Euro (PC). Total and annualized total costs were highest for MC, with PC being the significantly less costly option (*p* < 0.001).

**Conclusions:**

Within German healthcare, both initial and follow-up costs for post-restorations were considerable. Saving costs initially may, at least partially, be offset by more complications long-term.

**Clinical relevance:**

Dentists need to be aware that the placement of posts is not only initially costly but also comes with significant long-term costs for treating occurring complications. This should be communicated with patients and considered during treatment planning.

**Electronic supplementary material:**

The online version of this article (10.1007/s00784-020-03529-5) contains supplementary material, which is available to authorized users.

## Introduction

For endodontically treated teeth with extensive loss of coronal hard tissue, post placement is often performed to allow a subsequent core buildup. A number of previous investigations have assessed success and survival of post-restorations, showing limited annual failure rates of such restorations, at 0–2% per year. [1] Most of these studies, however, were performed mainly in university clinics.

For post-restorations, direct preformed metal (MP) or indirect cast metal (MC) posts, or non-metal glass-fiber (GF) posts can be employed. Theoretically, different post materials may come with different risks of root fracture [2, 3] and different failure rates. [4–7] However, clinical studies indicate only limited differences between different posts. [8] Notably, the placement costs for different posts may differ, with differences in laboratorial costs or luting efforts. Also, limited differences in risks of failure may, nevertheless, translate in substantial long-term costs depending if such failures are mainly re-cementations, renewals of the whole restoration, or extractions and tooth replacement. The coronal restoration (crown or direct composite restoration) may heavily affect initial costs and also long-term complications too, with direct post-retained composites (PC) probably being less costly, but possibly also less successful. [9]

A recent health economic study, employing a modeling approach based on data mainly from randomized trials, found only limited cost-effectiveness differences between different post-restorations. [10] Over a period of 26–28 years, and within the limitations of a modeling study, costs of 700–800 Euro occurred (i.e., annualized costs were around 27 Euro per restoration, including initial and re-treatment costs), with MP being slightly less costly, but also less effective than GF and MC. Overall, there is ambiguity as to the effectiveness and long-term costs of post-restorations. [11]

To resolve this, long-term, practice-based, prospective studies are needed, which allow to determine effectiveness and costs over relevant horizons, with sufficient depth of recording to allow economic analyses without the need to rely on models. The findings in these realistic settings are not only relevant for clinicians but also for payers and patients. A recent study on post-restorations from a practice-based network in Germany, for example, found much higher annual failure rates than most previous studies from other settings. [12] Based on this study, and considering long-term re-treatments experienced by different post-restorations, we performed a detailed health economic analysis to determine the costs for retaining teeth with post-restorations, and to evaluate factors, among them the type of post- and coronal restoration, which affect costs.

## Materials and methods

### Ethics, registration, and reporting

This prospective, practice-based cohort study did not need an ethical approval according to the local review board of the ethical committee of the Medical Chamber of Lower Saxony. Patients’ consent was not required given this being a retrospective evaluation of non-personal data. The study has been registered in the German Clinical Trials Register (DRKS00012938). Note, however, that the present analysis on long-term costs was not planned a priori, but is an auxiliary, exploratory analysis. Reporting of this study follows the STROBE [13] and CHEERS [14] guidelines.

### Data source

In this prospective, practice-based cohort study, involving eight general practices from a German dental practice–based research network (Arbeitskreis Zahnärztliche Therapie), patients receiving minimum one post-restoration were included. The cohort has been described in more detail before. [12] Briefly, patients receiving a post-restoration between June 2003 and November 2006 had been invited to participate. Patients needed to have had at least one incisor, canine, or premolar with a successfully completed root canal treatment, including an adequate root canal filling and no symptoms, as well as a coronal defect with three or more coronal surfaces missing. In cases when more than one tooth could have been included, only the first one in a convenience order was included in the study. For the present evaluation, only teeth which received a newly placed crown, bridge anchor, or coronal composite restoration were included (teeth receiving telescoping crowns, those where existing restorations were re-cemented including post placement, and those where composites were only placed as a temporary restoration were excluded). Per treating dentist, a maximum of 30 patients/teeth were to be included (cluster size). Decisions to maintain teeth and placed post-restorations were made according to the quality guidelines of the European Society of Endodontology [15].

### Data collection

For the present study, the following data were assessed from electronic case report forms: Initial and re-interventions, with materials for the post, the core, and the coronal restoration, as well as possible restorative re-treatments (re-cementations of the crown, the post, or renewal, or exchange of the complete restoration), endodontic re-interventions (non-surgical or surgical re-treatments), or extractions (due to fractures of the post, the tooth, or endodontic reasons). For teeth experiencing extractions, no data on prosthetic replacements for the teeth were provided, though, which may lead to costs being under-estimated. If repeated re-treatments were required, this was recorded, too. In addition, confounding variables like the name of the private practice and the dentist, the patient’s age and gender, the number of missing teeth, the tooth type, and if it was the last tooth in the dental arch or not, were recorded. Teeth and restorations were re-assessed via clinical and intraoral radiographic examinations in individual intervals. Examinations were performed by the dentist who placed the post-restoration, and while criteria for failure and treatment decisions had been discussed among the dentists beforehand, examiners were not calibrated; intra- or inter-examiner reliability data were not available.

### Setting, outcome, and horizon

The health economic analyses were performed in the context of German healthcare. Our outcome parameter was costs. The study’s horizon was determined by the follow-up of patients, with teeth either being extracted, or follow-up being concluded (censored). As teeth might have been retained after follow-up ended (censoring), our study did not aim to assess cost-effectiveness, but only costs per year of follow-up.

### Currency, price date, and discount rate

Costs were calculated in Euro 2018, assuming all patients have been provided with the post-restoration in 2018. This eases interpretation for today’s reader but may not capture the true costs borne by patients or the health insurances at the time. Future costs (i.e., those for follow-up treatments) were discounted at 3% per annum [16]. Discounting accounts for the lost opportunities when spending money now instead of later on.

### Perspective and estimation of costs

A public-payer’s perspective was chosen. Dental treatments in Germany are largely reimbursed by the statutory (public) insurance, with the majority of German patients (87%) being enrollees. [17] For these, nearly all costs are fully covered by the statutory insurance. For services not provided within the statutory insurance or for patients not publicly insured, private insurance laws apply. Within the present study, all costs (see below) fell into the statutory insurance.

Treatment costs within the statutory insurance are estimated using the statutory fee item catalog, Bewertungsmaßstab (BEMA). Laboratory costs were covered by the Bundeseinheitliches Leistungsverzeichnis (BEL-II). Details on items and cost estimation (using item-points being transformed into Euro) are provided within the appendix (Tables S1–4). We did not account for opportunity costs given that treatment and travel times had not been recorded.

Initial treatment costs included those for the initial clinical and radiographic examination, post placement, core buildup, a temporary post and a temporary crown if needed, and a crown being placed, assuming this to be a full metal crown for reasons of standardization. In case no crown, but a composite restoration was placed, costs for a four-surfaced incrementally placed restoration, including additional efforts for moisture control and matrix application, were applied. Moreover, material and laboratory costs for the models, dye and resin materials, metals if needed, and technical manufacturing of the restorations, were estimated. Re-treatment costs included those for assessment, radiographs if needed, re-cementation, crown or composite restoration renewal, post renewal using the same or a completely new coronal restoration, apicectomy, and extraction, with local anesthesia if needed.

### Analytical methods

Costs were calculated using a spreadsheet (Excel, Microsoft, Redmond, USA). Costs were discounted, and total and annualized costs covering initial and re-treatments estimated. We additionally assessed follow-up costs only. Association of total and annualized total costs with patient- and tooth-level parameters was evaluated via generalized linear mixed models (i.e., multivariable modeling), accounting for clustering and also accounting for follow-up. A reduced set of covariates was employed in a sensitivity analysis. Statistical analysis was performed using SPSS 22 (IBM, Armonk, USA). Visualization was done using the Python plotting library Matplotlib. [18]

## Results

A total of 139 post-restorations were included and followed over a mean ± SD (min.–max.) 7.1 ± 4.5 (0.2–13.7) years. The following post materials and the coronal restorations had been used: (1) MP plus crowns, *n* = 68; (2) GF plus crowns, *n* = 28; (3) metal or glass-fiber posts in combination with directly built up, post-retained composite restorations (PC, *n* = 20); and (4) MC, *n* = 23.

MC showed the highest initial treatment costs (557.23 Euro), but the least re-treatments (6/23, 26%), while PC showed lowest costs (203.52 Euro) but most re-treatments (11/20, 55%). Costs for MP/GF were initially similar (496.47/496.87 Euro), and both also showed similar re-treatments (24/68, 35%/10/28, 36%). The mean annual failure (re-treatment) rate was 5.2% (MC: 3.5%, MP: 4.6%, GF: 5.3%, PC: 10.3%). During follow-up, annual costs (SD) for MC, MP, GF, and PC were 7.12 (21.92), 10.58 (19.65), 10.42 (21.97), and 34.91 (83.34) Euro, respectively.

The results of initial and follow-up costs are total costs. These were the highest for MC, and lowest for PC; this was also applicable when total costs were annualized. Total follow-up costs, in turn, were highest for PC, and lowest for MC, also when follow-up costs were annualized (Table 1). Total costs did not differ greatly between genders, or age groups, and tended to be lower for individuals with more missing teeth. Tooth type and tooth position also did not greatly impact on total costs. Annualized total costs were lower in older individuals and those with more teeth, though. For follow-up costs, SDs were generally higher than the mean values, and no significant differences were identified by bivariate analysis (Table 1).

**Table 1:** Costs stratified according to post/restoration type, gender, age, missing teeth, and tooth type and position

			Total costs		Total costs, annualized		Total follow-up costs		Total follow-up costs, annualized	
			Mean	SD	Mean	SD	Mean	SD	Mean	SD
Post/restoration type	MC	23	591.66	92.70	171.36	190.63	34,43	92.70	7.12	21.92
	GF	28	526.37	103.28	146.12	151.29	39.98	82.01	10.42	21.97
	MP	68	548.31	132.88	141.75	158.98	56.15	126.11	10.58	19.65
	PC	20	361.81	180.51	135.65	131.89	143.63	168.07	34.91	83.34
Gender	Male	61	527.76	149.78	160.17	167.48	64.45	140.85	16.48	51.56
	Female	78	521.47	144.31	136.08	151.05	59.88	110.98	11.14	20.35
Age	< 55 years	83	515.32	152.86	164.54	176.28	59.98	138.55	14.19	45.95
	55 + years	56	537.44	136.07	120.14	123.99	64.70	101.34	12.44	18.66
Missing teeth	> 20	8	448.16	171.89	252.37	204.63	46.35	43.24	24.18	28.01
	11–20	35	585.45	161.55	120.48	130.43	105.34	167.63	25.83	65.23
	< 11	96	508.25	131.98	147.39	161.39	47.33	106.76	8.09	18.68
Tooth type	Incisor	26	507.74	141.34	146.28	174.96	46.95	107.72	6.31	13.40
	Canine	34	549.92	166.91	161.79	160.77	80.38	124.40	16.51	25.77
	Premolar	79	518.60	138.68	140.26	153.18	58.84	130.11	14.55	45.88
Last tooth in arch	No	127	525.68	146.45	145.93	159.48	63.38	128.29	13.50	38.52
	Yes	12	508.93	149.35	154.29	151.96	46.06	75.40	13.36	22.18

Generalized linear modeling found significant associations between covariates and total costs (Table 2, *p* < 0.001). Total costs differed significantly between post-restorations (*p* < 0.001, PCs were significantly less costly than crowned ones). Similarly, annualized total costs were significantly lower in PC than in MC (*p* < 0.001), if accounting for age, dental arch, tooth type, missing teeth, treating dentists, tooth position, and follow-up period as confounders. Annual costs also increased significantly with follow-up (Table 2).

**Table 2:** Association between total and annualized total costs and covariates. Coefficients (in Euro) and lower/upper confidence intervals (LCI/UCI) as well as levels of significance (*p*, in italics: *p* < 0.05) are provided

		Total costs				Total costs, annualized			
Parameter	Class	Coeff.	LCI	UCI	Sig.	Coeff.	LCI	UCI	Sig.
Post-restoration	PC	− *217*	*− 292*	*− 141*	*< 0.001*	*− 102*	*− 154*	*− 49*	*< 0.001*
	GF	− 51	*−* 110	9	0.097	*−* 10	*−* 51	32	0.642
	MP	− 65	*−* 134	4	0.067	*−* 41	*−* 89	7	0.094
	MC	Ref.				Ref.			
Gender	Female	− 8	*−* 50	35	0.722	*−* 10	*−* 40	18	0.490
	Male	Ref.				Ref.			
Age	per year	0	*−* 2	2	0.978	*−* 1	*−* 2	0	0.047
Missing teeth	> 20	− 2	*−* 122	119	0.961	*−* 57	*−* 139	27	0.184
	11–20	60	*−* 57	117	0.313	*−* 45	*−* 126	36	0.277
	< 11	Ref.				Ref.			
Tooth type	Premolar	9	*−* 46	65	0.745	*−* 11	*−* 49	28	0.593
	Canine	16	*−* 52	82	0.650	*−* 8	*−* 55	38	0.725
	Incisor	Ref.				Ref.			
Tooth position	Last tooth	*−* 23	*−* 115	70	0.632	*−* 37	*−* 101	26	0.249
	Not last tooth	Ref.				Ref.			
Follow-up	Per month	0	0	0	0.093	*− 3*	*− 3*	*− 2*	*< 0.001*

In a reduced model, including only the post-restoration type, the gender, age, and follow-up period, we found PC to be significantly less costly than MC again both when assessing total and total annualized costs. For the latter, age and follow-up period were also significantly associated, with lower costs, with each year of age and follow-up (Table S5).

**Fig. 1:**
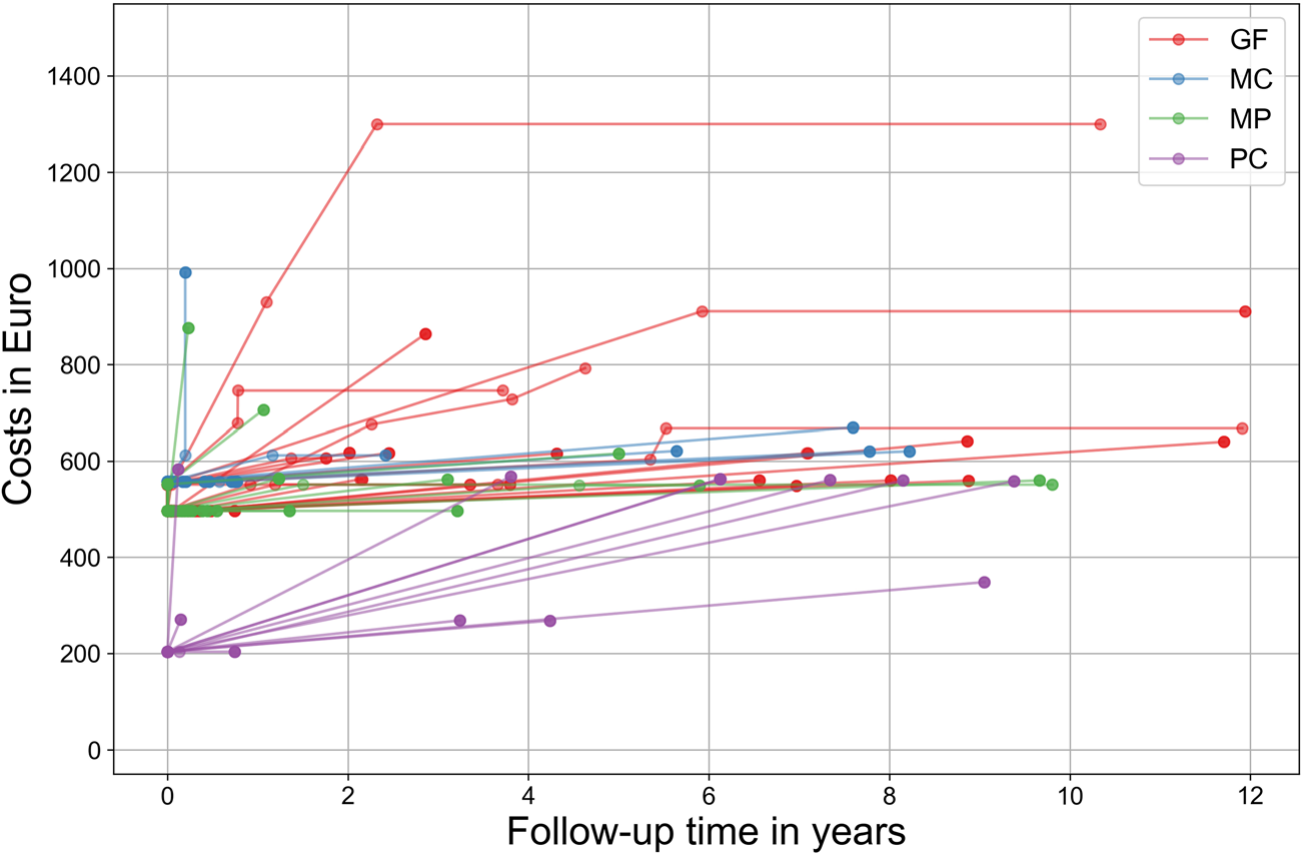
Cumulative costs (in Euro) were plotted over follow-up time (in years). Different colors indicate different post-restorations (red: GF, blue: MC, green: MP, purple: PC); each dot indicates a post-restoration

If cumulative costs were assessed, PC started with significantly lower costs, but the majority of PC then experienced costs for re-treatments (Fig. 1). However, even if accounting for these, total costs did only seldom exceed those for other post-restorations.

## Discussion

The present study assessed the costs of 139 post-restorations, placed and followed-up in a practice-based setting. Overall, the annual failure rate was, at 5.2%, rather high. Moreover, both the initial and follow-up costs were high and inversely correlated (higher initial costs came with lower costs during follow-up and vice versa). After a mean of 7 years, the initially least costly treatment, PC, nevertheless remained significantly less costly than the initially most expensive option, MC, despite requiring more re-treatments.

Our primary outcome (costs) was the result of both the initial costs and the need for re-treatments, i.e., the risk of failure. For post-restorations, as discussed, annual failure rates of around 2% have been recorded, [1] while some university-based studies also found annual failure rates of 4.6% [19] and 4.2%. [20] In one single university-based study, even up to 10% annual failure rates were observed. [21] Thus, the annual failure rates of this cohort in private practice environments are at the higher end of the range of failure rates. Nonetheless, it seems in line with previous studies, considering that differences in the study location (practice- vs. university-based), inclusion criteria (all teeth except molars vs. all teeth vs. only premolars), the included post materials (all types of posts vs. solely glass-fiber posts), the final restoration (solely crowns or bridge anchors vs. all types of restoration), and the treatment protocol (shared decision-making in in the present study vs. treatment decision following planned protocols in prospective studies) might influence failure rates. Overall, the yielded failure rates may be considered satisfying from a clinical perspective. [22]

This is the first practice-based study assessing long-term costs of post-restorations. Based on our findings, both initial but also re-treatment costs for post-restorations are significant. With annualized costs of 135–171 Euro in mean, retaining teeth via post placement may, overall, have only limited cost-effectiveness compared with alternative strategies, like tooth replacement. Note that these costs are also much higher than those estimated using a modeling approach, mainly built on data from randomized trials (with lower risks of failure), finding annualized cost of around 27 Euro. This may be partially explained by the longer horizon of the modeling study, where the high initial costs were distributed over a mean follow-up period of 26–28 years (decreasing them to around 20 Euro per tooth and year). The re-treatment costs in both studies were at around 7–10 Euro per year, i.e., similar, but it should be highlighted that in the modeling study, these included costs for prosthetic replacement of extracted post-teeth; this was not the case in the present study. Overall, it seems that post-restorations come with high initial and considerable re-treatment costs, being a rather costly and high-maintenance treatment strategy.

We further associated total and annualized total costs with a range of confounding variables. As discussed, post and also coronal restoration type were significantly associated with costs, especially if annualized costs were assessed (annualized costs are the more valid outcome parameter, as they reflect different follow-up periods, with higher costs being partially the result of longer follow-up periods, as demonstrated). Patients’ age, gender, number of missing teeth, and tooth type or location were not significantly associated with costs. Data on risks of restorative failure along genders are ambiguous, [12, 23] so we did not necessarily expect to identify significant associations here. Similarly, age has so far not been identified as a strong predictor for failure of post-restorations. We had expected the number of missing teeth to possibly affect the risk of failure and the costs, mainly due to unfavorable distribution of masticatory forces [24]. This was not the case. Previous studies showed that the longevity of posts was significantly influenced by the tooth type, with more complications in anterior teeth than molars, mainly due to biomechanical reasons [19, 25]. An association with costs may hence be expected. In the present study, however, molars were not included, which may explain the lack of any association, but also flags the need to interpret our estimated costs with caution (costs in molars may be somewhat lower).

This study is subject to a number of limitations. First, with a sample size of 139 teeth, it is most likely underpowered to detect association between costs and some covariates. Larger sample sizes or more balanced class distribution (e.g., only very few restorations were placed in the last teeth in each dental arch) may increase the power. Second, the setting, a practice-based network in Germany, and the study design, a prospective observational study, are prone to a number of biases. Dentists joining such networks may not be representative for all dentists in Germany, and neither may the care provided nor the patients treated. The dentists did not allocate treatments at random; it is conceivable that certain treatments (e.g., composites instead of crowns) may have been placed based on indications (e.g., perceiving poorer prognosis), so causal associations may not apply. The same dentist who provided the treatment also assessed its success during follow-up. Again, bias is likely here. Overall, the applicability of our findings may be limited when transferred to other settings, and caution is needed when assessing the found associations. The limited generalizability also applies to other types of post-crown types, for example double crowns retaining removal dentures, where costs (and longevity) will differ. Third, restorations in the present study were pragmatically classified according to both the post material and the coronal restoration. Notably, we merged glass-fiber and preformed metal posts into one group if they served to retain a direct composite restoration (PC), mainly as the PC group was of limited sample size. Stratifying it further would have increased uncertainty and decreased statistical power. Last, our cost estimation was built on fee items from the statutory insurance in Germany. These have been found to reflect the true direct medical costs to some degree, and studies using these yielded comparable results with studies from other settings, with other methods of cost estimation. [26–28] Nevertheless, cost estimates may not be fully transferable to other healthcare settings, and direct non-medical as well as indirect (opportunity) costs were not included at all. These have been found relevant if repeated treatment appointments are needed (incurring high travel costs and much time spent for traveling and treatment) and considering them has relevance from a societal perspective [26, 29].

## Conclusions

Within the limitations and generalizability of this study, both initial and follow-up costs for post-restorations were considerable. Saving costs initially may, at least partially, be offset by more complications long term. Comparative controlled studies should assess the cost-effectiveness of retaining teeth using post-restorations versus alternative treatment strategies. Exploring between-dentist variation in costs seems worthwhile, too.

## Electronic supplementary material

ESM 1 (DOCX 32 kb).

## Data Availability

Data used in this study can be made available if needed within data protection regulation boundaries.
