# Supplementary material for: Long-term costs of post-restorations: 7-year practice-based results from Germany
Source: Clin Oral Investig. 2020 Aug 28;25(4):2175–81. doi: 10.1007/s00784-020-03529-5 (PMC7966625; doi:10.1007/s00784-020-03529-5)
Supplement: Supplementary file 1 — (DOCX 32 kb). [file 784_2020_3529_MOESM1_ESM.docx]

Appendix Table S1: Costs per course of treatment

| **Course of treatment** | **Euro** | **Source** |
| --- | --- | --- |
| MP | 496.47 | Table S2 |
| GF | 496.87 | Table S2 |
| MC | 557.23 | Table S2 |
| PC | 203.52 | Table S3 |
| Recementation | 54.92 | [Schwendicke and Stolpe, 2017] |
| New crown | 379.37 | [Schwendicke and Stolpe, 2017] |
| New composite | 119.26 | Table S3 |
| New preformed metal post | 127.04 | Table S4 |
| New glas-fibre post | 127.44 | Table S4 |
| Apisectomy | 154.63 | [Schwendicke and Stolpe, 2017] |
| Extraction | 67.41 | [Schwendicke and Stolpe, 2017] |

Appendix Table S2: Items used to estimate costs for post-core crowns (MC, MP, GF).

| Treatment | Position BEMA/GOÄ/GOZ/L | Points or Euro | Number of treatments | Euro |
| --- | --- | --- | --- | --- |
| Clinical investigation | 01 | 18 | 1 | 19.44 |
| Radiographic assessment | GOÄ925a | 12 | 1 | 12.96 |
| Post^1^ | 18a / 18b | 50 / 80 | 1 | 46.49 |
| Core^2^ | 13b | 39 | 1 | 36.26 |
| Temporary post^3^ | 21 | 28 | 1 | 26.03 |
| Temporary crown | 19 | 19 | 1 | 17.66 |
| Recementation temporary crown | 24c | 7 | 1 | 6.51 |
| Crown full metal | 20a | 148 | 1 | 137.60 |
| Dental materials excl. post |  |  | 1 | 45.99 |
| Post, example. FRC Postec Plus, Henry Schein^2^ |  |  | 1 | 11.89 |
| Post, example. ParaPost Fiber Lux, Henry Schein^2^ |  |  |  | 12.29 |
| Subtotal |  |  |  | 334.80/ 335.20/ 340.57 |
| *Laboratory* | | | | |
| Situation model | 0010 | 6.64 | 2 | 13.28 |
| Used resin | 0023 | 14.04 | 1 | 14.04 |
| Single-tooth dye | 0051 | 10.64 | 1 | 10.64 |
| Occludator | 0120 | 9.74 | 1 | 9.74 |
| Metal post casting^3^ | 1050 | 54.99 | 1 | 54.99 |
| Full-metal crown | 1021 | 83.58 | 1 | 83.58 |
| Non-precious metal alloy | 9700 | 13.50 | 1 | 13.50 |
| Delivery | 9330 | 5.63 | 3 | 16.89 |
| Subtotal |  |  |  | 161.67/ 161.67/ 216,66 |
| **Total preformed metal post (MP)** |  |  |  | **496.47** |
| **Total glass-fibre post (GF)** |  |  |  | **496.87** |
| **Total metal cast post-and-core (MC)** |  |  |  | **557.23** |

^1^ Item 18a for preformed metal posts, item 18b for cast metal post-core

^2^ Applies only for preformed metal or glass-fibre posts.

^3^ Applies only for cast post-core.

BEMA points are transformed into Euro; one point equals 1.0802 Euro for non-prosthetic treatment items, and 0.9297 Euro for prosthetic treatment items (https://www.kzvb.de/fileadmin/user_upload/Zahnarztpraxis/Abrechnung/Punktwerte/pdf/Punktwerte_Quartal_1_2019.pdf).

Table S3: Items used to estimate costs for post-retained composites (PC).

| Treatment | Position BEMA/GOÄ/GOZ/L | Points or Euro | Number of treatments | Euro |
| --- | --- | --- | --- | --- |
| Clinical investigation | 01 | 18 | 1 | 19.44 |
| Radiographic assessment | GOÄ925a | 12 | 1 | 12.96 |
| Post | 18a | 50 | 1 | 46.49 |
| Post, example. ParaPost Fiber Lux, Henry Schein |  |  |  | 12.29 |
| Moisture control | 12 | 10 | 1 | 10.80 |
| Tooth separation and matrix application | 12 | 10 | 1 | 10.80 |
| Four-surfaced composite restoration | 13g | 84 | 1 | 90.74 |
| **Total PC** |  |  |  | **203.52** |

BEMA points are transformed into Euro; one point equals 1.0802 Euro for non-prosthetic treatment items, and 0.9297 Euro for prosthetic

Table S4: Replacement of a post

| Treatment | Position BEMA/GOÄ/GOZ/L | Points or Euro | Number of treatments | Euro |
| --- | --- | --- | --- | --- |
| Clinical investigation | 01 | 18 | 1 | 19.44 |
| Radiographic assessment | GOÄ925a | 12 | 1 | 12.96 |
| Post | 18a | 50 | 1 | 46.49 |
| Core | 13b | 39 | 1 | 36.26 |
| Post, example. FRC Postec Plus, Henry Schein |  |  | 1 | 11.89 |
| Post, example. ParaPost Fiber Lux, Henry Schein |  |  |  | 12.29 |
| **Total preformed metal post** |  |  |  | **127.04** |
| **Total glass-fibre post** |  |  |  | **127.44** |

Metal cast post-and-core restorations were always full-restoration renewal.

Table S5: Association between total and annualized total costs and covariates in a reduced model. Coefficients (in Euro) and lower/upper confidence intervals (LCI/UCI) as well as levels of significance (p, in bold: p<0.05) are provided.

|  |  | **Total costs** | | | | **Total costs, annualized** | | | |
| --- | --- | --- | --- | --- | --- | --- | --- | --- | --- |
| **Parameter** | **Class** | **Coeff.** | **LCI** | **UCI** | **Sig.** | **Coeff.** | **LCI** | **UCI** | **Sig.** |
| Post-restoration | PC | **-218** | **-295** | **-142** | **<0.01** | **-97** | **-150** | **14** | **<0.001** |
|  | GF | -48 | -109 | 12 | 0.115 | -10 | -51 | 31 | 0.634 |
|  | MP | -63 | -132 | 7 | 0.078 | -43 | -91 | 3 | 0.075 |
|  | MC | Ref. |  |  |  |  |  |  |  |
| Gender | Female | -13 | -55 | 30 | 0.565 | -10 | -39 | 19 | 0.513 |
|  | Male | Ref. |  |  |  |  |  |  |  |
| Age | per year | 0.7 | -0.7 | 2.1 | 0.344 | **-1.0** | **-2.0** | **-0.1** | **0.042** |
| Follow-up | per month | 0.4 | -0.1 | 0.8 | 0.055 | **-2.5** | **-2.8** | **-2.3** | **<0.001** |
|  |  |  |  |  |  |  |  |  |  |

References

Schwendicke F, Stolpe M: Cost-effectiveness of Different Post-retained Restorations. Journal of endodontics 2017;43:709-714.
